# Phenotyping for Drought Tolerance in Different Wheat Genotypes Using Spectral and Fluorescence Sensors

**DOI:** 10.3390/plants14142216

**Published:** 2025-07-17

**Authors:** Guilherme Filgueiras Soares, Maria Lucrecia Gerosa Ramos, Luca Felisberto Pereira, Beat Keller, Onno Muller, Cristiane Andrea de Lima, Patricia Carvalho da Silva, Juaci Vitória Malaquias, Jorge Henrique Chagas, Walter Quadros Ribeiro Junior

**Affiliations:** 1 Faculty of Agronomy and Veterinary Medicine, University of Brasília, Campus Darcy Ribeiro, Brasília 70910-970, DF, Brazil; soaresgf30@gmail.com (G.F.S.); agro.cristiane@gmail.com (C.A.d.L.); patriciacarvalhoagro@gmail.com (P.C.d.S.); 2 Federal Institute Goiano, Faculty of Agronomy, Campus of Posse-Fazenda Vereda do Canto, Posse 73900-000, GO, Brazil; lucas.felisberto@ifgiano.edu.br; 3 Forschungszentrum Jülich GmbH, 52428 Jülich, Germany; b.keller@fz-juelich.de (B.K.); o.muller@fz-juelich.de (O.M.); 4 Brazilian Agricultural Research Corporation—(EMBRAPA Cerrados), Planaltina 73310-970, DF, Brazil; juaci.malaquias@embrapa.br (J.V.M.); walter.quadros@embrapa.br (W.Q.R.J.); 5 Brazilian Agricultural Research Corporation (EMBRAPA Trigo), Planaltina 73310-970, DF, Brazil; jorge.chagas@embrapa.br

**Keywords:** gas exchange, NDVI, PRI, correlation, plant morphology

## Abstract

The wheat planted at the end of the rainy season in the Cerrado suffers from a strong water deficit. A selection of genetic material with drought tolerance is necessary. In improvement programs that evaluate a large number of materials, efficient, automated, and non-destructive phenotyping is essential, which requires the use of sensors. The experiment was conducted in 2016 using a phenotyping platform, where irrigation gradients ranging from 184 (WR4) to 601 mm (WR1) were created, allowing for the comparison of four genotypes. In addition to productivity, we evaluated plant height, hectoliter weight, the number of spikes per square meter, ear length, photosynthesis, and the indices calculated by the sensors. For most morphophysiological parameters, extreme stress makes it difficult to discriminate materials. WR1 (601 mm) and WR2 (501 mm) showed similar trends in almost all variables. The data validated the phenotyping platform, which creates an irrigation gradient, considering that the results obtained, in general, were proportional to the water levels. The similar trend between sensors (NDVI, PRI, and LIFT) and morphophysiological, plant growth, and crop yield evaluations validated the use of sensors as a tool in selecting drought-tolerant wheat genotypes using a non-invasive methodology. Considering that only four genotypes were used, none showed absolute and unequivocal tolerance to drought; however, each genotype exhibited some desirable characteristics related to drought tolerance mechanisms.

## 1. Introduction

The significant impacts on crop development and production caused by drought make this theme one of the most studied abiotic stresses [1]. However, in plants, water deficit tolerance is a complex feature, as many genes have evolved [2] to prevent it, making it difficult to improve breeding programs that seek material for drought tolerance. Considering that drought is a predictable effect due to climate change affecting yield [3], including the reproductive period [4] and terminal stress [5], the environmental aspect appears as the bottleneck to accelerating climate adaptation breeding, including the underdevelopment of specific analyzing methods/tools [6] and high-throughput sensors in breeding programs could be a shortcut for it. Several specific mechanisms in response to unfavorable environments have evolved in plant species throughout their evolutionary process, including growth inhibition, osmotic regulation, and detoxification [7,8].

Water restriction is responsible for significant morphological changes in plants. Physiological and biochemical dysfunctions in plants cause several changes in their cellular metabolism [9,10]. Morphological changes occur indirectly due to a series of physiological adjustments, such as osmotic adjustment, which involves the rapid and effective increase in solute concentration [11], stomatal closure, and a reduction in photosynthesis [12,13,14].

For wheat, considerable variation in water stress tolerance has been identified among cultivars, accessions, and related species, as well as a variation in the effect of water stress on physiological attributes in different regions [15,16].

Thus, the breeding program should consider identifying physiological characteristics responsible for drought tolerance as independent genetic loci that control yield and drought resistance. Therefore, practical and efficient methods that enable the identification of resistant individuals by analyzing their physiological activity during water deficit become valuable tools for plant breeding [17].

Based on this, it is noted that developing and improving protocols for drought tolerance assessment and its use in plant phenotyping may provide a better understanding of the traits related to this factor. With this knowledge, selection criteria can be established, as well as strategies for using these sources of resistance in plant breeding programs. Effective selection could make it possible to understand the complex relationships between genotype, environment, and phenotype, aiding breeding programs. In addition, genomics certainly would reach limited results without a trustworthy phenomics.

Recently, time-consuming and destructive physiological assessments have been used. Physiological evaluations using IRGA are accurate and non-destructive; however, they are laborious and time-consuming, making them unfeasible for breeding programs that utilize a large number of accessions.

Using efficient, non-destructive, and high-throughput sensors can make the selection of drought-tolerant genotypes more efficient and accurate. In addition, it may be an option for recovering precious individuals in segregating generations, which is not the case with destructive phenotyping. It also allows us to phenotype a large number of individuals through fast and automated analyses with very high yields, enabling scale expansion and the evaluation range of a new physiological tool for plant breeding, which can still be used for remote sensing [18,19].

PRI is an hyperspectral index used as a tool to detect variations in energy dissipation that occur when photosynthesis is reduced due to stress. It can be used to detect water stress even in the early stages, whereas NDVI only detects damage when it has already occurred [20]. Pérez-Ruiz et al. [21] obtained a linear correlation detected with a spectrometer. A statistically significant linear correlation was observed between the NDVI values obtained using the reference spectrometer [21].

New techniques using chlorophyll fluorescence, such as laser-induced transient fluorescence (LIFT), are emerging to allow easy and safe plant selection analyses for plant breeding [22,23]. The principle of LIFT is that the laser produced by a device excites chlorophyll in plants, promoting fluorescence emission [24]. The light emits high-frequency and short-duration flashlets during the fluorescence induction and relaxation [24]. Additionally, this technique offers a deeper understanding of the photochemical and non-photochemical processes that occur in the thylakoid membrane while also facilitating the study of characteristics related to the absorption and transfer of light energy in the electron transport chain [25].

Spectral indices have also provided useful information for monitoring different terrestrial surface environments [26,27] and have been increasingly disseminated, providing more accurate analyses of plant physiology. Among the various spectral indices, NDVI provides information on vegetation cover status, changes in land use and land cover patterns, photosynthetically active foliar density per unit area, leaf water content, and nutritional deficiencies in plants [14,28,29], which can be detected using NDVI standards. The PRI is another reflectance index and has been widely used to estimate light efficiency, reflecting plants’ photosynthetic performance [30,31].

In this context, it may be possible to differentiate resistant and stress-sensitive genotypes by determining the fluorescence values of chlorophyll a, NDVI, and PRI in evaluations throughout culture development. Thus, this work aimed to characterize wheat genotypes for drought tolerance, utilizing fluorescence and hyperspectral sensors as a potential tool to support the breeding program.

## 2. Results and Discussion

### 2.1. Morphological and Physiological Evaluations

All variables presented normal residuals, as determined by the Shapiro–Wilk test at a 5% significance level. Only leaf area and stalk length showed an interaction between genotypes and water regimes at 5% probability (Table 1 and Table 2).

For flag leaf area (FLA) in the interaction between genotypes within the water regime, PF020037 presented higher values, while the others remained statistically similar in WR1 and WR2 (Table 1). The most stressful treatments presented similar FLA. The water regime WR4 showed a lower flag leaf area, and in WR3, Brilhante showed a higher flag leaf area than PF020037. The FLA is considered a primary source of photoassimilates for grain filling due to the short distance between the ear and the flag leaf [32]. Thus, the area of the flag leaf can be considered an indicator of grain yield potential, as it has a predominant role in grain filling [33,34]. However, the higher FLA obtained in PF020037 did not increase grain yield (Table 2), indicating that this genotype made an excessive investment in green leaf area when subjected to 100% and 83% water replacement of evapotranspiration (WRE).

In unfolding the water regime within each genotype for FLA, the most stressful treatment did not differentiate the genotypes (Table 1). PF020037 appeared to be more sensitive to drought, as indicated by comparisons between WR3 and WR2 and WR1. Additionally, in WR4, for all genotypes, FLA was reduced by 50% compared to WR1. FLA reduction is a consequence of various physiological and morphological disorders in plants caused by water deficiency, resulting in reduced photosynthesis and negatively affecting growth, development, and grain yield [35]. Guendouz et al. [32] achieved a significant reduction in FLA under water stress conditions. According to these authors, water deficit decreases cell division, which in turn reduces turgor pressure. Consequently, cell expansion results in a smaller leaf area.

For peduncle length (PL), the unfolding of genotype interaction within each water regime, in WR1 and WR2, was observed only in PF020037, which presented the lower PL. For WR3 and WR4, all wheat genotypes had similar PL (Table 1). PL is a trait of high genetic heritability [36,37]; however, the results of the present study demonstrate that this trait can also be strongly influenced by environmental conditions, especially under water stress. This is because, in WR1 and WR2, the distinct behavior resulted from the genetic variability between genotypes. When they were subjected to water stress (WR3 and WR4), there was a limitation to the growth of the peduncle, resulting in a considerable reduction in its length, with a similar response observed among genotypes.

Regarding the water regimes within each genotype for PL, in general, all genotypes had lower values in WR4 (Table 1). However, Brilhante, BRS404, and PF080492 presented a significant reduction in PL of around 50% from WR1 to WR4, while in PF020037, the reduction in PL was 20%.

Mahpara et al. [38] stated that PL is a useful indicator of wheat production capacity in dry environments. Genotypes with longer stems have more carbohydrates stored to transfer to the seeds. In addition, in wheat, several non-leaf organs are photosynthetically active, and one of these is the exposed part of the stalk, which can assimilate CO_2_ when exposed to light [39,40]. According to Kong et al. [41], the exposed peduncle is a photosynthetically active organ that produces photosynthesis and, therefore, makes an important contribution to grain growth, particularly during the final stages of grain filling. The metabolism of the peduncle is one of the organs most susceptible to water stress [41]. The nonfoliar stomatal behavior contributes to photosynthesis and crop yield, especially in plants under water stress [42].

The analysis of simple effects for plant height was performed because the interaction was not significant (Table 3). The difference in height between genotypes was expected, as this is an intrinsic characteristic of each genotype and can be linked to lodging.

**Figure 1:**
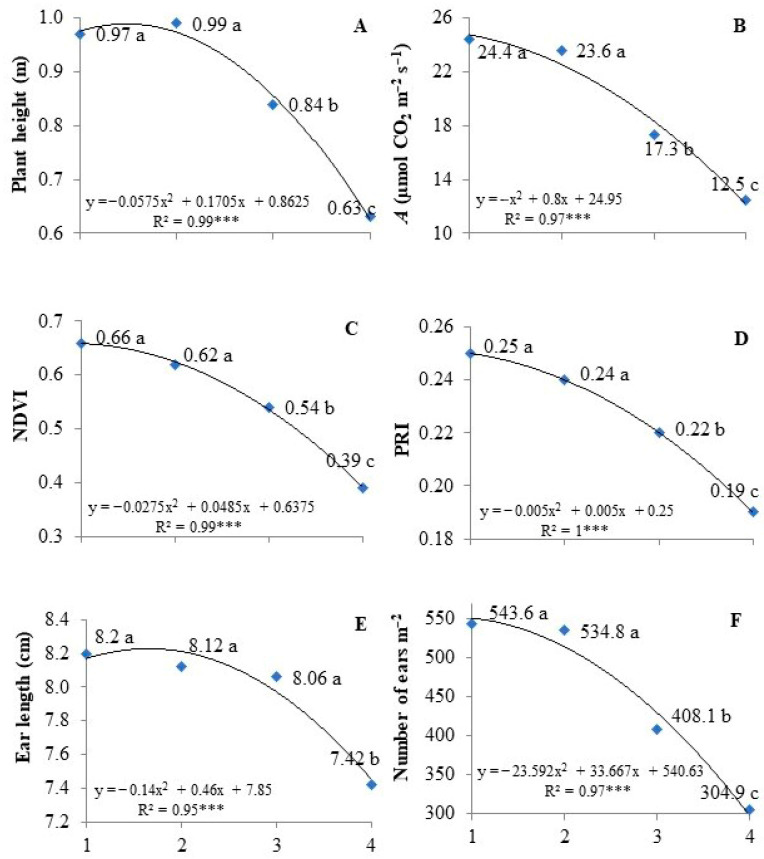
(**A**) Plant height, (**B**) photosynthesis, (**C**) NDVI, (**D**) PRI, (**E**) ear length (cm), and (**F**) number of ears of wheat genotypes under four water regimes using crop evapotranspiration replacement as a criteria (1–601 mm, 2–501 mm, 3–301 mm and 4–184 mm), during the winter period for the Brazilian Cerrado region. Data are a combination of four wheat genotypes. *** significant at 0.001%. Different letters in each graph indicate significative difference between water regimes at 0.001%.

Regarding the water regime factor, WR1 and WR2 were statistically similar and were higher than WR3 and WR4, respectively (Figure 1A). Plant height is a trait of high genetic heritability; however, it can be influenced by the environment, such as water stress [43,44]. Water is a key element in cell division, stretching, and differentiation. Under water deficit, cells grow sluggishly, resulting in stunted, malformed plants, and their phenological cycle is altered [45,46]. Phillips et al. [47] found optical sensor technology can be used to accurately estimate winter wheat tiller density.

Proline concentration (PRO) showed a significant interaction between genotype factors and water regimes. By unfolding the genotype interaction within each water regime, in WR1, all genotypes presented similar values (Table 1), indicating that under well-irrigated wheat genotypes do not alter proline content in the leaves. In WR2, the highest values were found in Brilhante (7.4 µmol g^−1^ fresh weight-fw), while the other genotypes presented similar values (Table 1). PF 020492 had a lower value under stress, indicating that this genotype does not use proline as a mechanism of drought tolerance. On the other hand, this response shows a rapid reaction of Brilhante to water stress conditions, as it significantly increased the proline concentration, with a slight decrease in the applied water (100 mm).

Wheat genotypes that accumulate higher proline concentrations can better tolerate water stress and higher growth [48,49] due to a greater capacity to maintain leaf water potential [50]. The increase in the concentration of compatible molecules, such as proline, maintains cell turgidity since the increase in osmotic pressure inside the cells maintains the flow of water in the plant, allowing its physiological processes to continue under conditions of low soil water potential [51].

In WR3, all genotypes showed significant increases in proline concentrations. However, Brilhante had the highest values of this osmoregulator (10 µmol g^−1^ fw). PF020037 and PF080492 presented the lowest proline concentrations (3.67 and 3.32 µmol g^−1^ fw, respectively). This result demonstrates that these genotypes are more sensitive to moderate water deficit.

Under severe water stress (WR4), the lowest values were found in PF080492, and the others presented similar values. This genotype has exhibited low concentrations of this amino acid (0.97 µmol g^−1^ fw) since WR2, and subsequently, a slight increase in proline concentration as water stress intensified. This response suggests that this genotype likely has low adaptation to water deficit, as the increase in proline concentration plays a crucial role in the protective antioxidant system, reducing oxidative damage and increasing tolerance to water stress in wheat plants [52,53]. Regarding the water regimes within each genotype, the lowest proline values were obtained in WR1 and WR2 in all genotypes. This is because the increase in the concentration of this amino acid occurs only under water limitation to mitigate the deleterious effects on physiological processes [9,54]. Thus, the increase in leaf proline concentration was only verified as the intensity of water stress was increased in WR3 and WR4. Proline is a solute highly sensitive to water stress conditions; therefore, determining this amino acid content in leaves provides an important parameter for selecting plants tolerant to environmental stress, especially water deficit [55,56].

Gas exchange parameters showed a significant interaction between genotypes and water regimes (Table 1), except for the net CO_2_ assimilation rate (A) (Table 3). For A, Brilhante presented higher values than PF020037, similar to genotypes BRS404 and PF080492 (Table 3). This distinct trend in photosynthesis between genotypes was already expected, as natural variation in photosynthetic capacity among wheat genotypes is frequently reported in the literature [57,58,59,60].

Sikder et al. [61] also reported high variation among six wheat genotypes in the net rate of CO_2_ assimilation. According to these authors, the variation observed was due to the derivation lines, indicating that even with evidence suggesting that the domestication of wheat resulted in materials with lower photosynthetic rates, there is genetic diversity for this trait. It is possible to increase the photosynthetic capacity of wheat cultivars.

WR1 and WR2 presented a higher net rate of CO_2_ assimilation and were statistically different from WR3 and WR4 (Figure 1B). These reductions can be attributed to the lower photosynthetic rate due to the damage to photosynthetic metabolism caused by water deficit [62], mainly due to the production of reactive oxygen species (ROS), which may damage the photosynthetic electron transport chain components and consequently reduce the photosynthetic capacity of plants [63,64].

In the unfolding interaction of gs, Ci, and E, all genotypes presented similar values in WR2 and WR4, except for gs in WR1. In WR3, a reduction of 300 mm of applied water in relation to WR1, Brilhante showed higher gs than PF020037 and higher Ci and E than the other wheat genotypes. In general, Brilhante can maintain higher net CO_2_ assimilation rates than PF020037, even with similar values of gs, Ci, and E. The higher A values found in WR3 by Brilhante in relation to PF020037 result from the higher gs, Ci, and E values obtained for this genotype (Table 1).

For gs, Ci and E, wheat genotypes generally presented similar trends as a function of water regimes, and WR1 and WR2 presented similar values, which were higher than those of WR3 and WR4, respectively (Table 1). These results were similar to the simple effect obtained in A (Figure 1B). However, BRS404 for gs and PF020037 for gs and E obtained higher values in WR1 than in WR2 (Table 1). Nevertheless, the decrease in stomatal conductance under mild water stress, as occurred in PF020037 and BRS404 in WR2 (100 mm reduction in water applied, compared to WR1), was not sufficient to significantly affect the rate of CO_2_ assimilation (Figure 1B).

BRS404 and PF020037 also obtained lower gs, Ci, and E in WR3 and were statistically similar to WR4 (Table 1). This response demonstrates their greater sensitivity to moderate water stress conditions (WR3), as they considerably reduced the values of these variables in this water regime. Thus, these genotypes exhibit a limited ability to maintain a photosynthetic rate under water stress conditions, which is crucial for preserving wheat growth and yield.

In water deficient conditions, plants close their stomata and reduce stomatal conductance to restrict water loss and reduce transpiration. As a consequence, decreased CO_2_ absorption occurs, reducing the photosynthetic rate and compromising the accumulation of photoassimilates in plants [65,66].

The maximum potential of photosynthetic capacity is rarely achieved in the field, even under favorable conditions. This occurs due to stomatal limitation resulting from limited soil water availability and a delay in stomatal response to changes in photosynthesis under fluctuating environmental conditions [65,66].

The cumulative rate of photosynthesis during plant growth and development is determinant for crop yield [67,68].

Therefore, under drought conditions, plant growth and yield reduction are usually related to the decrease in photosynthetic activity [69,70]. Thus, the gas exchange evaluation is a very useful tool in diagnosing the integrity of the photosynthetic apparatus, considering that it is a precise and non-destructive technique [66,71] and is strongly correlated with crop yield.

Maximum quantum yield of photosystem II (Fv/Fm) and effective quantum yield of photosystem II (Fv′/Fm′) were similar for all wheat genotypes within each WR, except for Fv′/Fm′ in WR3 (Table 2).

This different response between genotypes in WR3 for Fv′/Fm′ demonstrates a more intense inhibition of photosynthetic activity in photosystem II (PSII) for PF080492 compared to PF020037 (Table 2). This result agrees with those found in the gas exchange variables A, gs, Ci, and E (Table 1), demonstrating that the Fv′/Fm′ has high sensitivity in predicting the integrity of the photosynthetic machinery of wheat plants. Therefore, wheat genotypes that are more adapted to water stress conditions may be selected with the determination of Fv′/Fm′ since it estimates that photosynthesis’s quantum efficiency is easy to measure.

For the effect of water regimes within each genotype, in general, WR1 was statistically higher than WR4 in all wheat genotypes for Fv′/Fm′ and Fv/Fm (Table 2). This response suggests greater photoinhibition in plants under severe water stress since the reductions in the quantum yields of photosynthesis indicate significant damage to PSII [72].

Björkman and Demmig [73] determined the Fv/Fm ratio values in a large number of vascular species and found that healthy leaves of various species had Fv/Fm values around 0.832 ± 0.004. According to Krause and Weis [25], values below 0.80 cause strong photoinhibition of photosynthesis. Thus, WR1, WR2, and WR3 generally maintained the Fv/Fm close to this optimal value (0.83), unlike the Fv/Fm in WR4, which was 0.80.

PF020037 was the exception, which showed statistically similar values among all water regimes in Fv/Fm (Table 2). The conservation of Fv/Fm values as a function of the applied water regimes likely occurred due to genotype characteristics, indicating that most of the radiation was perhaps being utilized in the photochemical phase of photosynthesis. Therefore, PSII was not impaired due to water stress. The ability to maintain similar Fv/Fm values under water stress may indicate high efficiency in the use of radiation, possibly by carbon assimilation reactions [74].

However, Fv/Fm, which represents the efficiency of energy capture by PSII open centers [72], was less sensitive than Fv′/Fm′ in the evaluation of photosynthetic capacity, and this is due to Fv′/Fm′ quantifying the operational efficiency of the PSII. In addition, Fv′/Fm′ is more efficient for determining the variations in the quantum yield of photosynthesis compared to the Fv/Fm ratio [75]. This higher sensitivity of Fv′/Fm′ in relation to Fv/Fm to detect changes in photosynthetic capacity can be confirmed in the correlations between the gas exchange parameters.

Chlorophyll fluorescence has been widely used to study the photosynthetic capacity of plants. Besides being non-destructive, this technique allows for the qualitative and quantitative evaluation of the absorption and utilization of light energy by the photosynthetic apparatus [76]. In addition, chlorophyll a fluorescence is an effective method for evaluating photosynthetic performance, providing detailed information on the integrity of complex light collectors [77].

**Figure 2:**
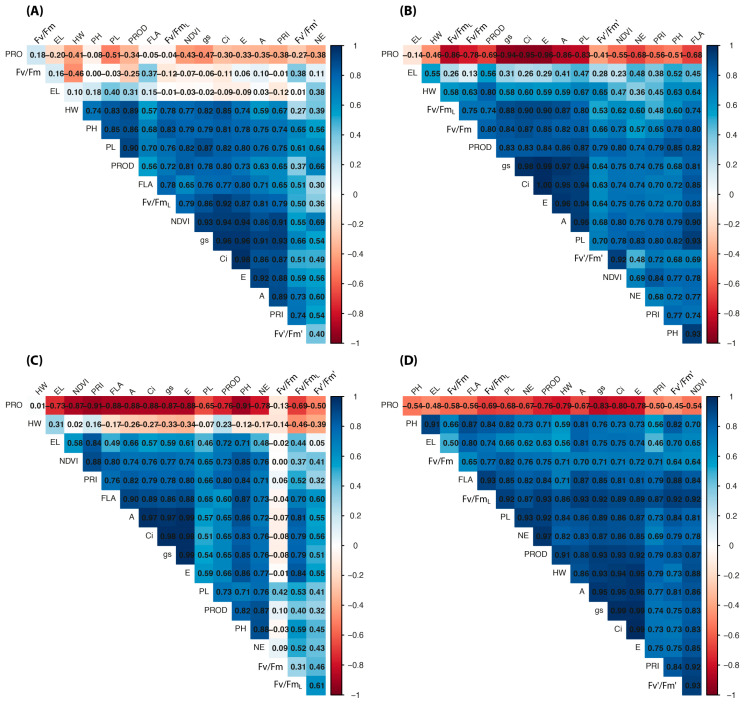
Correlogram of Pearson between morphophysiological traits (ear length, plant high, flag leaf area, ear length, ear number, peduncule length, hectoliter weight, thousand grain weight, yield, Irga measurements (photosynthesis, transpiration, internal carbon, stomatic conductance), proline, sensor index (NDVI, PRI), and fluoresce measurements) for (**A**) Brilhante, (**B**) BRS404, (**C**) PF020037, and (**D**) PF080492.

A wax layer on plants may influence normalized difference vegetation index (NDVI) and photochemical reflectance index (PRI) measurements used to detect drought stress in plants, as it frequently affects light reflectance, which is the basis for these vegetation index measurements, as noted by Khadka et al. [78], who found this in wheat, recommending caution when evaluating those genotypes. However, the results showed lower correlation using fluorescence for the waxy genotype PF020037 (Figure 2).

NDVI and PRI indices did not show an interaction between the studied factors; thus, each factor was presented separately. For NDVI, Brilhante, BRS404 and PF020037 genotypes obtained statistically higher values than PF080492 (Table 3). Vegetation indices are commonly used to estimate plant biophysical characteristics, mainly for leaf area index and biomass accumulation [79,80].

Therefore, it is possible to differentiate wheat genotypes by NDVI standards, as it is sensitive to identifying variations in plant biomass. This parameter has been effective in predicting wheat yield [81]. These results agree with the findings of Hazratkulova et al. [82] and Ramya et al. [83], who confirmed that it is possible to differentiate wheat genotypes through NDVI values for selecting genotypes resistant to abiotic stress.

By assessing the WRs as an isolated factor, WR1 and WR2 did not differ from each other and presented NDVI values higher than WR3 and WR4, respectively (Figure 1C). A higher NDVI value indicates that the plants have more chlorophyll and better development, leading to a higher productive potential. Plants under stress exhibit a decrease in chlorophyll absorption and a reduction in infrared reflectance due to changes in cell structure. This decrease is accompanied by a decline in chlorophyll absorption and a reduction in infrared reflectance, resulting from changes in cell structure, which in turn leads to an increase in red reflectance [84].

Gizaw et al. [85], who worked with wheat under three different water regimes, also obtained similar results to those presented in this work. The authors observed that NDVI values were higher in irrigated conditions and lower in dry conditions. Crusiol et al. [86] also found that NDVI values were higher in irrigated plants than in plants under water stress in soybean.

PF020037 showed higher PRI compared to the other wheat genotypes (Table 3). This likely occurred because PF020037 produces leaf wax, which likely occurred because PF020037 produces leaf wax, which is an intrinsic characteristic of this genotype. According to Holmes and Keiller [87], waxes are efficient reflectors of longer wavelength radiation, which may have overestimated both the PRI and the NDVI in P020037. The reflectance is influenced by both the biochemical elements of the leaf and morphological characteristics, such as cuticle waxes, which interfere with photon scattering [88,89].

Regarding the water regimes, PRI values in WR1 and WR2 were higher and statistically similar; followed by WR3 and WR4, respectively (Figure 1D). The PRI is sensitive to changes in leaf carotenoid pigments, which play a crucial role in photoprotection, as they dissipate excess energy before it can damage crucial role in photoprotection, as they dissipate excess energy before it can damage cellular structures [90]. This photoprotection is related to the defense of the photosynthetic apparatus against extremely reactive singlet oxygen that damages many cellular components [91]. Therefore, these pigments play a crucial role in preventing oxidative damage induced by water stress.

The PRI is based on the reversible changes in short-term xanthophyll pigment in plants under water stress [90]. These changes are linked to the dissipation of excess absorbed energy that cannot be processed through photosynthesis and, thus, lead to reduced light-use efficiency [30]. Therefore, PRI has been a very useful tool for estimating light efficiency, which in turn reflects the photosynthetic performance of plants [92].

Thus, PRI can serve as a tool to indicate water stress in plants. Moreover, this index can facilitate early stress detection, aiding decision-making in commercial crops and enabling the selection of stress-tolerant materials in breeding programs [93].

In this sense, the identification of physiological characteristics that relate to yield can be used for plant selection, as the productivity of a plant is the product of intercepted solar energy and fixed CO_2_ over a given period. However, evaluations of photosynthetic metabolism using IRGA have limitations for rapid screenings, as they can only evaluate a few plants or even one or two leaves per plant, making it a costly practice. Thus, hyperspectral sensors using the NDVI and PRI proved to be a quicker and more efficient method for evaluating physiological traits, as they enabled faster analysis of photosynthetic performance in plants, which significantly contributes to the process of selecting plants tolerant to water stress.

It was expected that Fv′/Fm′ responses obtained from LIFT (Fv′/Fm′_L_) were similar to those obtained from IRGA (Fv′/Fm′), as found by other authors when evaluating both measurements simultaneously [18,94]. However, in this work, Fv′/Fm′ from IRGA was between these two methods for quantifying fluorescence.

Flagella et al. [95] found slight increases in Fm in wheat during the vegetative phase, followed by a reduction during flowering, decreasing the Fv′/Fm′ ratio since Fo remained constant. In this work, Fv′/Fm′ measurement with IRGA was evaluated 15 days before Fv′/Fm′ in the grain filling phase, in which there is intense chlorophyll degradation and disassembly of the photosynthetic apparatus due to photoassimilate remobilization to grains [96].

The yield components, number of ears, and ear length did not show the interaction between genotypes and water regimes. The simple effects indicated that PF080492 obtained the highest values for these two variables (Table 3). In Figure 1E, WR1, WR2, and WR3 did not differ from each other, but these were statistically higher than WR4 for ear length. The number of spikes (Figure 1F) was similar between WR1 and 2, which were higher than WR3 and WR4, respectively. The data in Figure 1A–F demonstrate a clear effect of water stress and the morphological data or plant growth and, thus, validate the indices obtained (PRI and NDVI).

Thousand-grain weight (TGW), hectoliter weight (HW), and yield showed a significant interaction between genotypes and WRs (Table 1). The evaluation of genotypes within each regime for TGW, Brilhante, was superior to the other genotypes in WR1. In the WR2, WR3, and WR4 regimes, PF080492 generally had the lowest average. For the effect of WRs within each genotype, WR1 was statistically similar to WR2 but superior to WR3 and WR4 in the Brilhante and PF080492 genotypes. BRS404 and PF020037 presented similar values among the regimes WR1, WR2, and WR3. In BRS404, all genotypes differed from WR4. However, for PF020037, W1000 in WR3 was statistically similar to WR4 (Table 1).

In the unfolding interaction for HW, the effect of genotypes within WRs was overall lower than that of PF020037 in WR1 and WR2. In contrast, in the more stressed regimes, WR3 and WR4, TGW was similar among wheat genotypes (Table 3).

Regarding WRs within each genotype, all genotypes presented higher HW values in WR1 than in WR4. However, BRS404 presented HW similar to WR3 and lower values in WR4.

Wheat HW values typically range from 70 to 85 kg hl^−1^ but may be higher or lower due to environmental conditions [97,98]. Thus, the higher the HW value, the better the quality of wheat flour. Therefore, the ability of BRS404 to maintain high HW values until moderate water stress (WR3) conditions, similar to that found in the off-season in the Brazilian Cerrado, is an interesting feature to be considered for the selection of materials intended for this cultivation period, because the HW is an important variable for commercialization since it indirectly indicates the quality characteristics of grains.

Therefore, the ability of BRS404 to maintain high values of HW until the moderate water stress condition (WR3), a condition similar to that found in the second crop of the Brazilian Cerrado, is an interesting feature to be considered for the selection of materials for this cultivation, because the HW is an important variable for marketing since it indirectly indicates the characteristics of grain quality. For yield, in the unfolding interaction for wheat genotypes within each WR, PF020037 produced fewer grains than the other genotypes in WR1 (Table 1). In WR2, this genotype showed a similar yield to BRS404 and Brilhante. PF080492 in this WR produced 1300 kg ha^−1^ more than the other genotypes (Table 1).

In the most stressed regimes, WR3 and WR4, there was no statistical difference among all genotypes in grain yield (Table 1). This result indicates that, under severe water stress, although some genotypes performed better in certain biometric or physiological variables (Table 1), they were insufficient to promote higher productivity. However, in mild stress (WR2), PF080492 proved to be an interesting material in terms of productivity.

The four genotypes generally presented higher values in the regimes WR1 and WR2 compared to WR4 (Table 1). For BRS404, WR1 expressed the highest yield compared to WR3. PF080492 performed better on WR1 and WR2, and WR3 was statistically different from WR4. For PF020037, the WR1, WR2 and WR3 presented similar yield, and with higher values than WR4. Although PF020037 generally had the lowest yield, it was the only genotype to maintain similar yield values until the WR3 water regime. It is important to note that only four genotypes do not represent all genetic variability, even for the Brazilian Cerrado and rainfed conditions. Time-consuming measurements prevent the use of a large number of genotypes.

### 2.2. Pearson Correlation and Principal Component Analysis

Pearson correlation was performed for each wheat genotype (Figure 2A–D). For Brilhante, there was a positive correlation with most variables, except for proline, flag leaf area, Fv/Fm, Fv′/Fm′, and ear length. BRS404 showed a positive correlation with all variables, except for ear length, and a negative correlation with proline (r = −0.69 *). For PF020037, most variables correlated positively with productivity, except for proline, which presented a negative correlation (−0.76 **) and did not correlate with productivity. The variables Fv′/Fm′_L_, Fv/Fm, and Fv′/Fm′ also did not correlate with productivity. Wheat genotype PF080492 showed a positive correlation with all variables except for proline, which had a negative correlation with productivity (−0.76 **). Flag leaf area showed a positive correlation with yield, with an exception for Brilhante. The other genotypes showed correlations of 0.60 for PF020037 and 0.84 for PF08492. PF020037 is waxy and this may be linked to miscalculations in LIFT sensors (Figure 2). Plants with a larger leaf area present a larger photosynthetically active area, which transfers a greater amount of photoassimilates for grain filling, thereby increasing crop yield [33].

The responses of PH were similar to those found for yield (Table 1), and the correlation between these variables ranged from 0.71 (PF08492) to 0.86 (Brilhante) (*p* < 0.01). Similar results were obtained by Modarresi et al. [99] with thermal stress in wheat; the authors obtained a correlation of 0.85 between PH and yield. PH is an important variable for the establishment of grain yield as stalk reserves are an essential source of carbohydrates for complete grain filling, especially under water stress conditions when remobilization is increased [100].

Plant height (PH) presented a positive correlation of 0.54 (*p* < 0.01) with yield in semi-dwarf wheat. Several studies have demonstrated a positive correlation between these variables, and cultivars had higher yields [101,102]. However, as water stress intensified, proline accumulation in leaf tissues had a negative correlation to yield, with the exception of Brilhante, which did not show a correlation with this variable. These results indicate that wheat plants exposed to water deficit increase proline accumulation, but there is a significant reduction in yield (Table 2). These results agree with a literature review by Serraj and Sinclair [103] who stated that most published articles indicate no effect or a negative influence of increased osmolyte concentration on crop yield. Thus, the findings in this study support the hypothesis that proline plays an important role in osmoprotection; however, it does not reflect increases in wheat yield under water stress.

In this study, a high positive correlation was found between gas exchange variables (A, gs, Ci and E) and yield for all wheat genotypes, with the exception of PF020037 (0.65 for A, gs and Ci), and Brilhante (0.63 for A and 0.73 for gs) (Table 4).

The Fv/Fm_L_ showed a strong correlation with gas exchange for all wheat genotypes. The Fv/Fm correlated with gas exchange for BRS404 and PF080492 but did not correlate with gas exchange for PF020037.

The differences found between genotypes in leaf area and plant height (Table 1) are reflected in variation in the NDVI. This evidence is confirmed by the correlations obtained between NDVI and the PH and FLA for all wheat genotypes.

PRI values found in WR3 and WR4 are possibly related to the reduction in light use efficiency, which consequently affected the photosynthetic performance of wheat genotypes. The correlations obtained between PRI and the gas exchange variables confirm this assumption, wherein A, gs, Ci, and E presented high correlation rates with PRI for all wheat genotypes. According to Magney et al. [93], air temperature, stomatal conductance, and vapor pressure deficit are mechanically related variables are related to PRI. This suggests that the PRI is sensitive to estimating these variables.

The number of ears (NE) and ear length (EL) were correlated with yield (*p* < 0.01) for all wheat genotypes (Table 4). However, for EL, only PF080492 (0.63) and PF020037 (0.75) correlated with yield. This result demonstrates that this production component has a limited influence on wheat grain yield, agreeing with Ojha et al. [104], who did not find a correlation between ear length and yield in wheat genotypes.

In contrast, NE was a significant variable for selecting more productive genotypes, as it showed a strong correlation with yield for all wheat genotypes (Table 4). According to Khan and Naqvi [105], NE can be used to select high-yield wheat at different irrigation levels. The main component responsible for regulating grain yield in response to environmental factors is NE [106].

Thousand-grain weight showed a correlation with yield only for Brilhante (0.51) and PF080492 (0.77). The correlation of TGW with yield in wheat is described by several authors [107,108], and this response occurs because TGW may respond to modest changes in genetic or environmental factors, thus acting with a fine adjustment of grain yield [106].

The HW response as a function of WRs was similar to that found in wheat yield (Table 1), which characterizes it as an important variable for selecting more productive genotypes. This can be confirmed by the strong correlation between HW and yield, except for PF020037. These results are in agreement with those found by Dogan [109], who found a correlation of 0.76 between HW and yield in *Triticum durum*.

**Figure 3:**
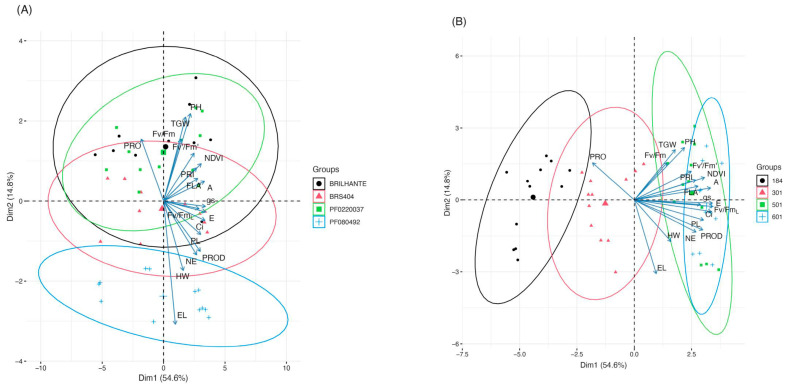
Exploratory analysis of the principal components for morphophysiological variables (ear length, plant high, flag leaf area, ear length, ear number, peduncule length, hectoliter weight, thousand grain weight, yield, gas exchange measurements (A, gs, E and Ci), proline, sensor index (NDVI and PRI), and fluoresce measurements (Fv/Fm, Fv′/Fm′ and Fv/Fm_L_) for (**A**) PCA compares genotypes, and (**B**) PCA compares irrigation levels. The vectors of the variables projected onto the graphs indicate the magnitude and direction of their contribution to the separation between groups. The length of the vectors reflects the intensity of their influence on the principal components, while their orientation highlights the multivariate differences between the evaluated conditions. The ellipses drawn around the experimental groups represent the intragroup dispersion based on the covariance of the data. The center of each ellipse corresponds to the centroid of the respective group, representing the average position of the observations. The orientation and size of the ellipses indicate the internal variability of each group: more compact ellipses suggest greater homogeneity within the observations.

An exploratory analysis of principal components (PC1 and PC2) was generated to investigate the relationship between morphophysiological evaluations and wheat genotypes and water regimes (Figure 3A,B). The distribution of variables showed a variance of 54.6% for PC1 and 14.8% for PC2, with a cumulative variance of 69.4%. Four wheat genotypes were evaluated, and each was represented by a set of replicate observations, showing morphophysiological variability. These components were created to discriminate the effects of water regimes and wheat genotypes. PC1 is related to PROD, PL, Ci, E, gs, Fv/Fm_L_, FLA, NDVI, PRI, and A. These variables were collinear, indicating a potential positive effect on growth and yield. PRO showed a vector orthogonal to most of the other variables, indicating partially independent behavior. NDVI, PRI, Pl, and Ci are highly correlated, suggesting that they are excellent candidates for key performance indicators. Figure 3A shows that the biplot reveals how the genotypes are distributed in the space of the main components, that is, how they are grouped based on the combinations of the original variables. The arrows represent the variables, while the points represent individual observations (plants or averages by genotype). Multivariate analysis by PCA showed clear differences in the performance of the four genotypes evaluated. The Brilhante genotype stood out for its alignment with the most desirable traits of physiological efficiency and productivity. In contrast, PF080492 was more aligned with variables associated with stress and low efficiency. The genotypes BRS404 and PF0220037 occupy intermediate positions, with specific nuances of morphology and photochemical efficiency.

In contrast, larger ellipses indicate greater heterogeneity, possibly associated with the different conditions imposed by the treatments. The distribution of the vectors and the separation of the ellipses demonstrate that the analyzed variables play a significant role in differentiating between the cultivar groups and irrigation regimes, providing insights into the physiological and spectral responses under each evaluated scenario. The results validated the platform and sensors; however, new and more diverse materials should be included to serve as selection tools in plant breeding.

## 3. Material and Methods

**Figure 4:**
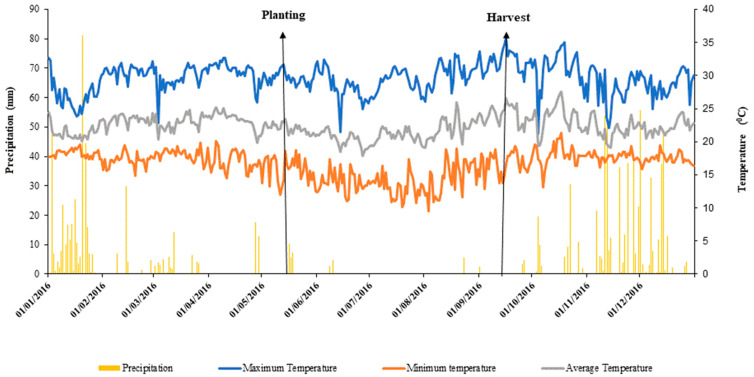
Rainfall (mm), maximum, minimum and average temperature (°C) in the experimental area in 2016.

The experiment was conducted in 2016 under a no-tillage system between June and September in the experimental area of Embrapa Cerrados, located in Planaltina DF, Brazil, with geographical coordinates 15°35′30″ S and 47°42′30″ W, 1000 m asl. The climate in the region is Aw, typical of savannas with two well-defined seasons: one dry and cold (autumn and winter) and another hot and humid (spring and summer), according to the Köppen classification [110]. The climatic conditions during the experiment are presented in Figure 4.

### 3.1. Soil Characterization

The soil is classified as typical Oxisol [111]. Before the experiment was installed, the area had been cultivated with wheat for ten years in the winter, using the same cultivars selected for the present study. The chemical characterization of the soil in the 0–20 cm layer, sampled before the installation of the experiment, showed the following results: pH (H_2_O) = 6.36; organic matter = 21.6 g dm^−3^; P (Melich) = 7.23 mg dm^−3^; K, Ca, Mg, H + Al, and C.T.C.: 0.35; 3.01; 1.72; 3.47 and 8.55 cmol dm^−3^, respectively.

### 3.2. Experimental Design and Genotypes Characterization

The experimental design was randomized blocks in a split-plot scheme with three replications. The plots comprised four wheat genotypes: Brilhante, BRS404, PF080492, and PF020037. The subplots corresponded to the water regimes (WR) (601 mm, 501 mm, 301 mm and 184 mm, named WR1, WR2, WR3, and WR4, respectively), which corresponded to 100%, 83%, 50%, and 30% of crop evapotranspiration replacement. Each experimental unit represented 1 m^2^. Genotype characteristics are as follows: Brilhante, a drought-tolerant rainfed biotype material; BRS404, a wheat cultivar launched in 2015, suitable for rainfed cultivation in Central Brazil; PF080492, classified as a rainfed biotype material in Southern Brazil, but in the Midwest region it has demonstrated suitability for rainfed agriculture; PF020037 is a line developed for rainfed agriculture and has as a relevant feature the presence of intense waxing on leaves and stalks, the natural mechanism of drought tolerance [112], although it has a tendency for lodging. Wheat genotypes were sown on 1 June 2016, using a tractor-traction experimental plow seeder with eight rows of 0.17 linear meters apart. The number of seeds was calculated to achieve a stand of 90 plants per meter, taking into account the results of the previous germination test.

### 3.3. Levels of Irrigation

Irrigation was homogeneous in all genotypes until 30 days after emergence. After this period of crop establishment, the line source methodology [113] was adapted, which was modified by introducing an irrigation bar with sprinklers of varying water flow rates, with the flow decreasing from the center to the end of the bar. The different sprinklers produced a decrease in water gradient from the central area of the bar (considered the ideal depth) towards the edge of the experiment, which allowed to obtain the water regimes.

**Figure 5:**
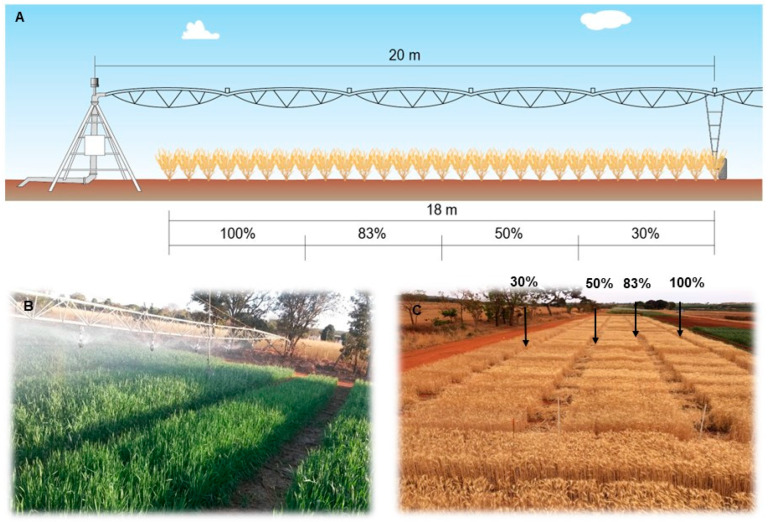
Scheme of the irrigation bar showing the size of and crop replacement of evapotranspiration (**A**); Irrigation bar in the field experiment showing the gradient of irrigation (**B**); Subplots of 1 m^2^ with 100%, 80%, 50% and 30% of crop replacement evapotranspiration after harvesting wheat genotypes (**C**).

Water regimes were applied with a sprinkler irrigation bar model 36/42 (IrrigaBrasil, Pinhais, PR, Brazil), 20 m wide on each side, connected to the TurboMaq 75/G.B. reel with adjustable speed. Figure 5 shows the irrigation bar and subplots of wheat genotypes irrigated and after harvesting.

During the uniform irrigation phase, 134 mm of water was applied from 1–30 June 2016 (including rainfall of 7.5 mm). The water regimes accumulated during the 105 days of the wheat cycle were 601 mm, 501 mm, 301 mm and 184 mm, corresponding to 3, 7, 11 and 15 m from the beginning of the bar, respectively. Each experimental unit had an area of 1.02 m. No irrigated controls were excluded because plants failed to survive (see Figure 4 for rainfall data). The water regimes were calculated based on crop evapotranspiration using the monitoring program in the Cerrado [114].

### 3.4. Morphophysiological Evaluations

On 6 August 2016, in full bloom of wheat genotypes, morphophysiological evaluations were performed in the subplot useful area, considered 1 m in each water regime and in the four central lines. Plant height and leaf area were evaluated in 10 plants per replication. The leaf area was measured using the LAI-2000 photoelectric meter, LICOR. Peduncle length (PL) was determined with a measuring tape, graduated in cm, in 10 plants per replication collected manually at harvest.

Proline concentration (μmol g^−1^) was evaluated in the flag leaf (four plants per replication) using the method of Bates et al. [115]. For the determination of proline content, 0.5 g of leaves was ground with liquid nitrogen, and 10 mL of 3% sulfosalicylic acid was added. The material was subsequently filtered through Whatman filter paper No. 2. The filtrate was added to test tubes with screw caps, containing 1.5 mL of extract, 3.0 mL of water, 0.1 mL of glycine, 2.0 mL of ninhydrin acid, and 2.0 mL of glacial acetic acid. The mixture was kept in a water bath (100 °C) for one hour for the development of color. Then, the test tubes were placed in an ice bath for 10 min to stop the reaction. The chromophore extraction was performed by adding 4 mL of toluene, forming a biphasic mixture, after vigorous stirring for 10 s in a vortex. After resting, the aqueous phase was aspirated and transferred to a glass cuvette for quantification of proline levels in the leaves using a spectrophotometer (SP-2000 U.V., Tecnal, Piracicaba, SP, Brazil), adjusted to 520 nm.

Gas exchange was evaluated in 15 replicates per treatment using a portable open-flow gas exchange system (IRGA-LAI-6400XT; LI-COR Inc., Lincoln, NE, USA), between 9:00 and 13:00 h, under irradiance of 1200 µmol photons m^−2^ s^−1^ and an internal CO_2_ concentration of 400 µmol mol^−1^. The net CO_2_ assimilation rate (A), stomatal conductance (gs), CO_2_ concentration in the substomatal cavities (Ci), and transpiration rate (E) were evaluated.

The maximum quantum yield of photosystem II (Fv/Fm) was calculated using the formula Fv/Fm = (Fm − F0)/Fm, according to Maxwell and Johnson [116]. Fv′/Fm′ gives the effective quantum yield of photosystem II′ = (Fm′ − F0′)/Fm′, according to Genty et al. [75]. The determination of the maximum fluorescence (Fm) and basal fluorescence (F0) were evaluated with the dark-adapted leaf, and the same parameters were collected with the light-adapted leaf, namely, Fm′ and F0′. These variables were obtained from the IRGA (infrared gas analyzer).

A terrestrial wheeled platform containing fluorescence and hyperspectral sensors was used. Fv′/Fm′_L_ data were obtained from the reflectance and hyperspectral sensors. For using a constant actinic light, the intensity of the blue LIFT LED in DC mode was calibrated using a quantum sensor (LI-190R, LI-COR, Inc.) at a distance of 0.6 m [24]. The photochemical reflectance index (PRI) and the normalized difference vegetation index (NDVI) were obtained. The light-induced fluorescence (LIFT) fluorescence sensor yielded the effective quantum yield of Photosystem II (Fv/Fm) using the same formula shown for Fv/Fm obtained from IRGA.

Through a spectrometer (STS-VIS, Ocean Optics), with a spectral range of 400–800 nm, and a blue LED (445 nm excitation source) installed in the LIFT instrument, raw digital values from the spectrometer output were used to derive vegetation indices, including the photochemical reflectance index (PRI) and the normalized difference vegetation index (NDVI). These spectral indices were calculated as follows: NDVI = (R800 − R640)/(R800 + 640) and PRI = (R531 − R570)/(R531 + R570). The data with poor signal-to-noise ratios were eliminated.

The NDVI was calculated as the ratio and normalization of indices, thus determined by the difference between the two bands called LIFT and near-infrared, divided by their sum. IR corresponds to the near-infrared radiant flux (750 nm), and R corresponds to the red radiant flux reflected from the visible region (550 nm).

$$NDVI=\left(\frac{IR-R}{IR+R}\right)\times 100$$

For the calculation of the PRI, the spectral reflectance in the visible range was used at wavelengths of 531 nm and 570 nm, representing R_1_ and R_2_, respectively.

$$PRI=\frac{\left(R_{1}-R_{2}\right)}{\left(R_{1}+R_{2}\right)}$$

The experiment was harvested manually on 13 September 2016. During this period, the following yield components were determined: ear length (EL), number of ears m^−2^ (NE), hectoliter weight (HW), thousand-grain weight (TGW), and yield. The EL was determined with a graduated ruler in centimeters, and ten ears were randomly evaluated for each experimental unit. The NE was determined by directly counting the harvested ears, excluding the edge of each plot, and extrapolated to m^2^. HW, TGW, and yield were evaluated according to BRASIL [117].

### 3.5. Statistical Analysis

Statistical analysis was performed using analysis of variance at a 5% significance level, as determined by the F-test, and the means were compared using Tukey’s test. The variation sources were wheat genotypes (plots), water regimes (subplots), and their interactions. Data showing no significant interactions, including the simple effects of wheat genotypes and water regimes, were presented.

The statistical model was adjusted using SAS, (version 9.4) Proc Mixed, employing the restricted maximum likelihood (REML) method. The data were also submitted to the correlation test between all variables using Past software (version 4.13). The correlation intensity rating for *p* < 0.05 was considered very strong (r = 0.91 to 1.00), strong (r = 0.71 to 0.90), medium (r = 0.51 to 0.70), and weak (r = 0.31 to 0.50), as cited by Guerra and Livera [118].

Principal component analysis was performed in R (version 4.3.3). Before the PCA, Bartlett’s sphericity test was performed, which showed a value of χ^2^ = 2265.637 (*p* < 0.001), indicating significant correlations between the variables. The KMO index was 0.83, indicating that the sample’s adequacy was classified as “good”.

PCA was performed with standardized variables. The first two main components were used for graphical interpretation, covering 70.56% of the total variance. The observations were plotted in a biplot, with 95% confidence ellipses shown for each genotypic group.

## 4. Conclusions

The data validated the phenotyping platform, which creates an irrigation gradient, considering that the results obtained, in general, were proportional to the water levels. The similar trend between sensors (NDVI, PRI, and LIFT) and morphophysiological plant growth and crop yield evaluations validated the use of sensors as a tool in selecting drought-tolerant wheat genotypes using a non-invasive methodology. Proline appears to be a mechanism for drought tolerance, considering that it is opposite to the parameters that grow with irrigation. PF080492 appears to be the most diverse genotype, with BRS 404 as an intermediate and Brilhante and PF020037 as the most similar. LIFT reduced the efficiency for yield for waxy genotype (PF020037). Comparing the different genotypes, there was none with absolute and unequivocal tolerance to drought.

## Figures and Tables

**Table 1:** Flag leaf area (FLA–cm^2^), peduncle length (PL–cm), proline content in flag leaf (PRO–µmol g^−1^ fw), stomatal conductance (gs–mol m^−2^ s^−1^), CO_2_ concentration in the substomatal cavities (Ci–µmol m^−2^ s^−1^), and transpiration rate (E) of four wheat genotypes under four water regimes.

Genotype	WR1–100%	WR2–80%	WR3–50%	WR4–30%
		FLA		
Brilhante	15.6 ± 4.64 Ab	14.7 ± 2.01 Ab	14.9 ± 3.85 Aa	6.7 ± 2.77 Ba
BRS404	15.0 ± 1.33 Ab	15.9 ± 0.80 Ab	13.0 ± 0.30 ABa	8.7 ± 0.81.Ba
PF080492	14.4 ± 1.36 Ab	17.6 ± 1.55 Ab	13.3 ± 0.61 ABa	8.0 ± 0.58 Ba
PF020037	21.6 ± 3.84 Aa	23.6 ± 1.10 Aa	12.6 ± 2.36 Ba	9.0 ± 1.94 Ba
		PL		
Brilhante	38.4 ± 0.95 Aa	33.2 ± 2.64 Bab	28.1 ± 1.47 Ca	19.4 ± 2.79 Da
BRS404	34.3 ± 2.22 Aa	32.2 ± 1.34 Ab	23.3 ± 2.01 Ba	16.9 ± 0.38 Ca
PF080492	35.8 ± 2.50 Aa	37.8 ± 5.02 Aa	27.5 ± 2.94 Ba	19.9 ± 0.48 Ca
PF020037	25.9 ± 0.11 Ab	25.6 ± 2.36 Ac	25.8 ± 2.01 Aa	20.6 ± 0.38 Ba
		PRO		
Brilhante	1.01 ± 0.19 Ca	7.4 ± 1.12 Ba	10.9 ± 0.58 Aa	6.65 ± 0.53 Ba
BRS404	1.02 ± 0.18 Ba	1.89 ± 0.37 Bb	6.13 ± 0.28 Ab	5.18 ± 1.22 Aa
PF080492	0.93 ± 0.09 Ca	0.97 ± 0.12 BCb	3.32 ± 0.55 Ac	2.60 ± 0.51 ABb
PF020037	0.72 ± 0.31 Ca	1.35 ± 0.55 Cb	3.67 ± 0.06 Bc	5.99 ± 1.89 Aa
		gs		
Brilhante	0.44 ± 0.03 Ab	0.38 ± 0.04 Aa	0.24 ± 0.09 Ba	0.11 ± 0.05 Ca
BRS404	0.55 ± 0.04 Aa	0.46 ± 0.05 Ba	0.15 ± 0.01 Cab	0.11 ± 0.03 Ca
PF080492	0.44 ± 0.00 Ab	0.47 ± 0.02 Aa	0.21 ± 0.07 Bab	0.10 ± 0.03 Ca
PF020037	0.53 ± 0.01 Aab	0.40 ± 0.06 Ba	0.14 ± 0.05 Cb	0.11 ± 0.03 Ca
		Ci		
Brilhante	277.1 ± 4.88 Aa	269.3 ± 18.02 Aba	249.1 ± 48.01 Ba	177.5 ± 39.73 Ca
BRS404	298.6 ± 2.66 Aa	288.8 ± 3.29 Aa	202.1 ± 1.48 Bb	187.8 ± 20.65 Ba
PF080492	292.8 ± 5.30 Aa	291.3 ± 1.06 Aa	227.1 ± 27.42 Bab	191.9 ± 13.36 Ca
PF020037	290.1 ± 2.71 Aa	271.4 ± 12.62 Aa	198.4 ± 24.38 Bb	193.8 ± 12.07 Ba
		E		
Brilhante	6.1 ± 0.49 Aa	5.9 ± 0.12 Aa	5.4 ± 1.52 Aa	3.2 ± 1.17 Ba
BRS404	7.2 ± 0.08 Aa	6.4 ± 0.14 Aa	3.1 ± 0.06 Bb	2.7 ± 0.67 Ba
PF080492	7.1 ± 0.03 Aa	6.8 ± 0.28 Aa	4.2 ± 1.05 Bb	2.4 ± 0.65 Ca
PF020037	7.0 ± 0.46 Aa	6.1 ± 0.51 Ba	3.0 ± 0.82 Cb	2.5 ± 0.53 Ca

Means followed by the same uppercase letters in the rows and lowercase letters in the columns for each variable do not differ from each other, as determined by the Tukey test at *p* < 0.05. WR1 to WR4 corresponded to 100%, 80%, 50%, and 30% of evapotranspiration replacement, respectively.

**Table 2:** Effective quantum yield of photosystem II Fv′/Fm′), maximum quantum yield of photosystem II (Fv/Fm), effective quantum yield of photosystem II obtained from LIFT (Fv/Fm_L_), thousand-grain weight (TGW), hectoliter weight (HW), and yield (kg ha^−1^) of four wheat genotypes under four water regimes.

Genotype	WR1–100%	WR2–80%	WR3–50%	WR4–30%
		Fv′/Fm′		
Brilhante	0.49 ± 0.1 Aa	0.52 ± 0.05 Aa	0.53 ± 0.01 Aab	0.50 ± 0.04 Aa
BRS404	0.55 ± 0.02 Aa	0.56 ± 0.02 Aa	0.49 ± 0.04 ABab	0.46 ± 0.03 Bab
PF080492	0.49 ± 0.05 Aa	0.48 ± 0.05 Aa	0.43 ± 0.06 Ab	0.36 ± 0.03 Bb
PF020037	0.49 ± 0.07 Aa	0.47 ± 0.07 Aa	0.55 ± 0.08 Aa	0.47 ± 0.10 Aa
		Fv/Fm		
Brilhante	0.83 ± 0.02 ABa	0.84 ± 0.01 Aa	0.82 ± 0.00 BCa	0.81 ± 0.01 Ca
BRS404	0.83 ± 0.01 Aa	0.82 ± 0.00 Aa	0.82 ± 0.01 Aa	0.80 ± 0.02 Ba
PF080492	0.83 ± 0.00 Aa	0.83 ± 0.00 Aa	0.82 ± 0.01 Aa	0.80 ± 0.01 Ba
PF020037	0.83 ± 0.01 Aa	0.83 ± 0.00 Aa	0.82 ± 0.01 Aa	0.82 ± 0.00 Aa
		Fv′/Fm′_L_		
Brilhante	0.59 ± 0.03 Aa	0.59 ± 0.04 Aa	0.57 ± 0.04 Aa	0.45 ± 0.05 Ba
BRS404	0.57 ± 0.02 Aa	0.55 ± 0.01 Aa	0.49 ± 0.02 Bab	0.48 ± 0.06 Ba
PF080492	0.56 ± 0.00 Aa	0.58 ± 0.01 Aa	0.53 ± 0.01 ABab	0.48 ± 0.01 Ba
PF020037	0.54 ± 0.02 Aa	0.53 ± 0.04 ABa	0.47 ± 0.02 Cb	0.48 ± 0.05 BCa
		TGW		
Brilhante	3.7 ± 0.11 Aa	3.12 ± 0.13 Aba	3.3 ± 0.01 Ba	2.9 ± 0.09 Ca
BRS404	3.4 ± 0.06 Ab	3.3 ± 0.0.11 Aab	3.3 ± 0.20 Aa	2.9 ± 0.13 Ba
PF080492	3.3 ± 0.08 Ab	3.1 ± 0.11 Ab	2.7 ± 0.12 Bb	2.4 ± 0.10 Cb
PF020037	3.4 ± 0.10 Ab	3.3 ± 0.12 Aab	3.4 ± 0.06 Aba	3.1 ± 0.28 Ba
		HW		
Brilhante	81.3 ± 1.3 Aa	80.3 ± 1.3 Aba	79.4 ± 0.92 Aba	78.1 ± 0.35 Ba
BRS404	82.6 ± 1.04 Aa	81.6 ± 2.12 Aa	81.6 ± 1.01 Aa	79.0 ± 0.25 Ba
PF080492	83.0 ± 0.31 Aa	81.6 ± 0.21 Aba	79.4 ± 0.51 Bca	78.1 ± 0.50 Ca
PF020037	77.1 ± 2.33 Ab	77.4 ± 2.95 ABb	79.7 ± 0.25 Ba	77.2 ± 0.61 Ba
		PROD		
Brilhante	4111.4 ± 1178.61 Aa	3582.7 ± 660.40 Ab	2858.3 ± 228.52 Ba	1078 ± 106.64 Ca
BRS404	4582.9 ± 583.54 Aa	3757.9 ± 587.85 ABb	3110.5 ± 267.46 Ba	1550.1 ± 354.86 Ca
PF080492	4921.5 ± 1010.84 Aa	5108.1 ± 730.02 Aa	2675.3 ± 171.75 Ba	1169.0 ± 101.08 Ca
PF020037	2858.4 ± 476.87 Ab	2752.9 ± 669.89 Ab	2595.1 ± 250.59 Aa	1298.6 ± 106.23 Ba

Means followed by the same uppercase letters in the rows and lowercase in the columns for each variable do not differ from each other, according to the Tukey test at *p* < 0.05. WR1 to WR4 corresponded to 100%, 80%, 50% and 30% of evapotranspiration replacement, respectively.

**Table 3:** Simple effect of plant height (PH-m), net CO_2_ assimilation rate (*A*-µm CO_2_ m^−2^ s^−1^), normalized difference vegetation index (NDVI), photochemical reflectance index (PRI), number of ears m^−2^ (NE), and ear length (EL-cm) of four wheat genotypes under four water regimes.

Genotype	PH	*A*	NDVI	PRI	NE	EL
Brilhante	0.94 ± 0.10 a	21.0 ± 5.67 a	0.57 ± 0.10 a	0.22 ± 0.03 b	432.8 ± 77.29 b	7.24 ± 0.46 b
BRS404	0.85 ± 0.11 b	19.2 ± 5.24 ab	0.55 ± 0.10 a	0.22 ± 0.02 b	430.0 ± 78.29 b	7.77 ± 0.37 b
PF080492	0.72 ± 0.08 c	19.1 ± 4.83 ab	0.50 ± 0.16 b	0.22 ± 0.03 b	511.0 ± 88.01 a	9.26 ± 0.73 a
PF020037	0.92 ± 0.12 a	18.4 ± 6.63 b	0.59 ± 0.11 a	0.25 ± 0.02 a	417.5 ± 70.28 b	7.55 ± 0.49 b

Means followed by the same letters in the column for each variable do not differ from each other by the Tukey test at 5% probability.

**Table 4:** Loads of the different variables associated with the principal component (PC) of water regimes and wheat genotypes.

Variable	PC1	PC2
PH	0.610	0.607
FLA	0.774	0.158
PRO	−0.507	0.431
A	0.927	0.138
gs	0.949	−0.037
Ci	0.937	−0.135
E	0.953	−0.076
Fv/Fm_L_	0.605	−0.053
Fv/FmA	0.692	0.333
Fv/FmB	0.396	0.430
NDVI	0.854	0.260
PRI	0.748	0.231
PL	0.841	−0.233
EL	0.267	−0.857
NE	0.750	−0.374
TGW	0.499	0.583
HW	0.446	−0.484
PROD	0.832	−0.350

## Data Availability

The data presented in this study are available on request from the corresponding author.
